# Aggressive surgery could overcome the extent of initial peritoneal dissemination for advanced ovarian, fallopian tube, and peritoneal carcinoma

**DOI:** 10.1038/s41598-020-78296-0

**Published:** 2020-12-04

**Authors:** Kyoko Nishikimi, Shinichi Tate, Ayumu Matsuoka, Makio Shozu

**Affiliations:** Department of Gynecology, Chiba University Graduate School of Medicine, 1-8-1, Inohana, Chuou-ku, Chiba city, Chiba 2608677 Japan

**Keywords:** Medical research, Oncology

## Abstract

We examined whether the extent of initial peritoneal dissemination affected the prognosis of patients with advanced ovarian, fallopian tube, and peritoneal carcinoma when initially disseminated lesions > 1 cm in diameter were removed, regardless of the timing of aggressive cytoreductive surgery. The extent of peritoneal dissemination was assessed by the peritoneal cancer index (PCI) at initial laparotomy in 186 consecutive patients with stage IIIC/IV. Sixty patients underwent primary debulking surgery and 109 patients underwent neoadjuvant chemotherapy followed by interval debulking surgery. Seventeen patients could not undergo debulking surgery because of disease progression during neoadjuvant chemotherapy. The median initial PCI were 17. Upper abdominal surgery and bowel resection were performed in 149 (80%) and 171 patients (92%), respectively. Residual disease ≤ 1 cm after surgery was achieved in 164 patients (89%). The initial PCI was not significantly associated with progression-free survival (PFS; *p* = 0.13) and overall survival (OS; *p* = 0.09). No residual disease and a high-complexity surgery significantly prolonged PFS (*p* < 0.01 and *p* = 0.02, respectively) and OS (*p* < 0.01 and *p* ≤ 0.01, respectively). The extent of initial peritoneal dissemination did not affect the prognosis when initially disseminated lesions > 1 cm were resected.

## Introduction

Majority of ovarian, fallopian tube, and peritoneal carcinoma have already spread to the peritoneal cavity beyond the pelvis upon diagnosis. Among the several assessment tools for the extent of peritoneal dissemination^1–6^, peritoneal cancer index (PCI) is precise and reproducible for the assessment of the location and size of lesions in 13 abdominopelvic regions^1,2^. It has been universally used for the assessment of the prognosis or surgical resectability of gastrointestinal carcinomas. Some reports have shown that PCI is also one of the prognostic factors for ovarian cancer^4,6–9^. A negative correlation has been reported between PCI and complete resection rate^10^, which is the most significant prognostic factor for ovarian cancer^11,12^.

Whether aggressive cytoreductive surgery overcome the extent of peritoneal dissemination remains debatable^3,13–15^. Aggressive surgery is necessary to achieve complete resection in patients with a high PCI score. However, in practice, aggressive surgery is not often performed for patients with a high PCI score at primary debulking surgery (PDS). Only 22% patients with high disease burden received high-complexity surgery in the Gynecologic Oncology Group 182 study^3^. In addition, complete cytoreduction rate in patients treated with PDS was approximately 20–50% even in highly experienced centers in which aggressive surgery is performed^16–18^, except for a few^7,19^. Perioperative death or severe perioperative complications in patients with a high PCI score could occur when aggressive surgery with PDS was performed^18^.

The treatment option for patients who could not achieve complete resection with PDS is neoadjuvant chemotherapy (NACT) followed by interval debulking surgery (IDS). It is unclear whether aggressive surgery overcomes the extent of dissemination for the patients treated with IDS. Usually, aggressive surgery is not performed during IDS in patients with a high initial PCI score before initiation of NACT^18^ because many initially disseminated tumors become invisible after NACT, and only visible tumors can be resected during IDS. Therefore, initially disseminated tumors were not resected in such cases. However, our previous study showed that lesions > 1 cm in diameter before NACT administration may harbor microscopic disease even though the initially disseminated tumor becomes invisible after NACT^20^. The previous study observed that the median progression-free survival (PFS) was longer in patients who underwent aggressive surgery with resection of lesions measuring > 1 cm before NACT than in those who underwent resection of only visible lesions during IDS^20^. Therefore, we concluded that initially disseminated tumors that could not be resected during IDS contributed to poor prognosis in patients with a high PCI score.

We hypothesized that aggressive surgery with resection of the initial > 1 cm dissemination would overcome the high PCI score by selecting PDS or NACT followed by IDS depending on whether cytoreduction to no residual disease is achievable at initial laparotomy before starting treatment. In this study, we examined whether PCI affected the prognosis of patients with International Federation of Gynecology and Obstetrics (FIGO) stage IIIC/IV ovarian, fallopian tube, and peritoneal carcinoma when initially disseminated lesions > 1 cm were resected, regardless of the timing of cytoreductive surgery.

## Results

### Patients' characteristics and correlation with the initial PCI (Table 1)

**Table 1:** Patients' characteristics and correlation with the initial PCI.

Variables	n = 186	PCI median [IQR]	*p* value
Age, median [IQR]	62 [51–70]	17 [10–22]	
Primary site			
Ovary	100 (54%)	15 [8–20]	< 0.01
Fallopian tube	74 (40%)	18 [14–27]	
Peritoneal	12 (6.5%)	19 [15–22]	
Performance status			
0	39 (21%)	16 [7–21]	0.20
1	83 (45%)	18 [11–22]	
2	46 (25%)	17 [11–22]	
3	18 (10%)	15 [13–18]	
FIGO stage			
IIIC	101 (54%)	16 [10–22]	0.78
IV	85 (46%)	17 [11–22]	
Histology			
High-grade serous	148 (80%)	17 [13–22]	< 0.01
Non high-grade serous	38 (20%)	11 [6–20]	
Low-grade serous	3 (2%)		
Clear	11 (6%)		
Endometrioid	10 (5%)		
Mucinous	3 (2%)		
Others	11 (6%)		
Timing of cytoreductive surgery			
Primary	60 (32%)	8 [5–16]	< 0.01
Interval	109 (59%)	19 [15–22]	
No debulking surgery	17 (9%)	22 [18–27]	
Surgical outcome			
No residual disease	148 (80%)	16 [9–21]	< 0.01
Residual disease 0.1–1 cm	16 (9%)	20 [16–24]	
Residual disease > 1 cm	22 (12%)^a^	22 [16–26]	
Surgical complexity score^b^, median [IQR]	12 [8–15]		
Low (1–3)	26 (14%)^a^	20 [7–25]	< 0.01
Moderate (4–7)	28 (15%)	8 [5–13]	
High (8–18)	132 (71%)	18 [13–22]	

PCI: Peritoneal Cancer Index, FIGO: International Federation of Gynecology and Obstetrics, IQR: Interquartile range.

^a^Including the 17 cases in which interval debulking surgery could not be performed because of disease progression during neoadjuvant chemotherapy.

^b^The score calculated based on complexity and number of surgical procedure performed reported by Aletti et al^13,21^.

The median initial PCI was 17 (minimum 2, maximum 31, first to third quartile range 10 to 22). The initial PCI was significantly associated with primary site, histology, timing of cytoreductive surgery, residual disease, and surgical complexity score. The initial PCI was not associated with performance status and FIGO stage.

Sixty patients (32%) underwent PDS. One hundred and nine patients underwent IDS after NACT. Seventeen patients did not undergo IDS because of disease progression during NACT (no debulking surgery). The median initial PCI of the patients who underwent PDS, IDS, and no debulking surgery were 8, 19, and 22, respectively. The initial PCI of the patients who underwent PDS was significantly lower than that of the patients who underwent IDS (*p* < 0.01).

### Surgical procedure and outcome

Of the 186 patients, 149 patients (80%) underwent upper abdominal procedure, including right diaphragm resection (*n* = 145) and splenectomy with or without distal pancreatectomy (*n* = 98), and 171 patients (92%) underwent bowel resection, including rectosigmoid colon (*n* = 163), large bowel other than the rectosigmoid colon (*n* = 90), and small bowel (*n* = 20). The surgical complexity score had a median value of 12 and was significantly higher in the patients who underwent IDS than in those who underwent PDS (13 vs 10, *p* < 0.001).

Overall, cytoreductive surgery achieved no residual disease in 148 patients (80%), residual disease measuring 0.1–1 cm in 16 (9%), and residual disease > 1 cm or no debulking surgery in 22 (12%). Among the 60 patients who underwent PDS, cytoreductive surgery was associated with no residual disease in 47 (78%), residual disease measuring 0.1–1 cm in 11 (18%), and residual disease > 1 cm in 2 patients (3%). Among the 109 patients who underwent IDS, cytoreductive surgery was associated with no residual disease in 101 (93%), residual disease measuring 0.1–1 cm in 5 (5%), and residual disease > 1 cm or no debulking surgery in 3 patients (3%).

The 30-day and 3-month mortality rates after cytoreductive surgery were 0% and 0.005%, respectively.

### Residual disease and surgical complexity score in each of the 3 groups of PCI (Fig. 1)

**Figure 1:**
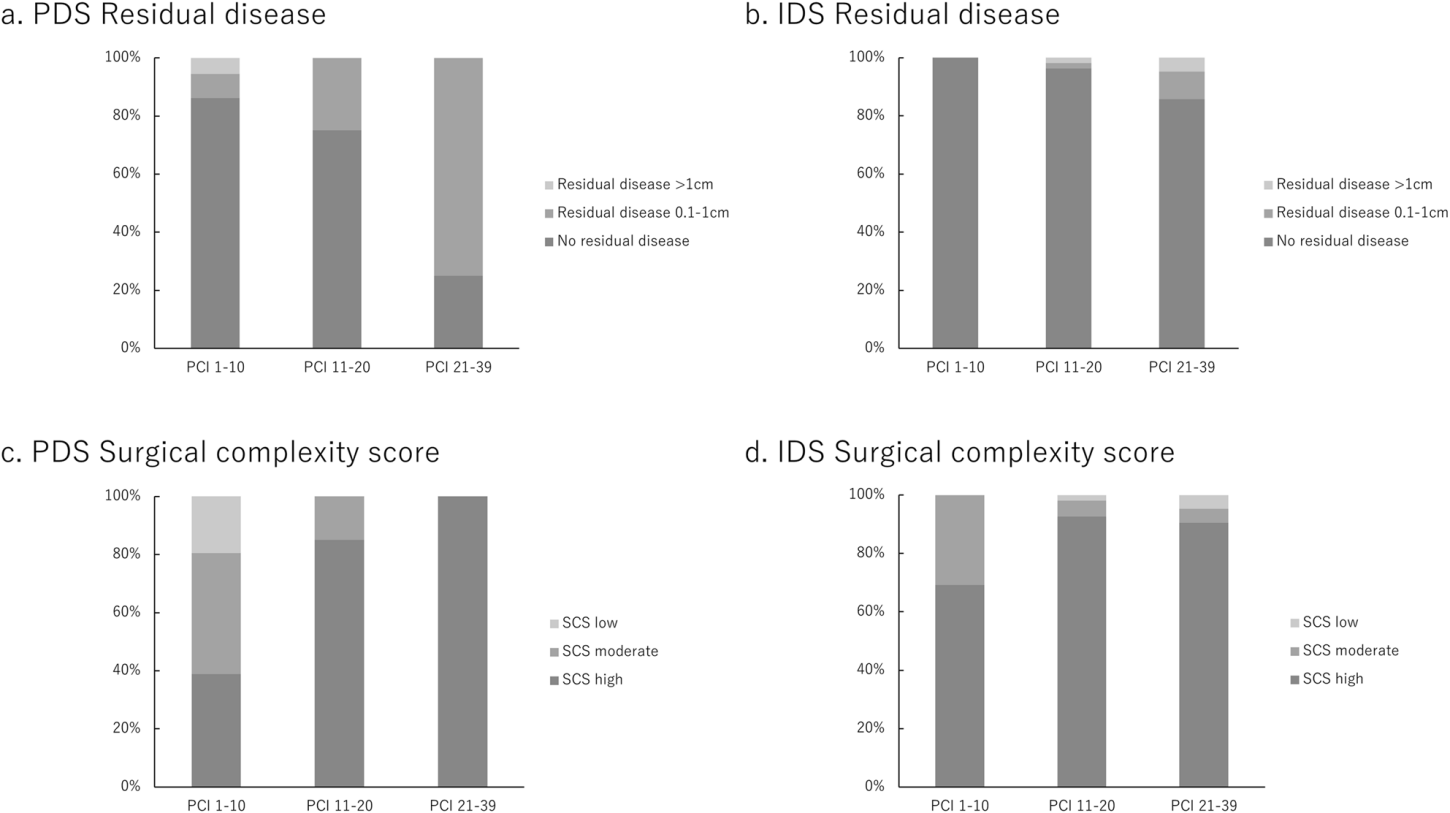
Residual disease and surgical complexity scores in subgroups of patients with PCI scores 1–10, 11–20, and 21–39. (a) Residual disease in patients who underwent PDS. (b) Residual disease in patients who underwent IDS. (c) Surgical complexity score in patients who underwent PDS. (d) Surgical complexity score in patients who underwent IDS. PDS: primary debulking surgery, IDS: interval debulking surgery, PCI: peritoneal cancer index, SCS: surgical complexity score.

Distribution of residual disease in 60 patients who underwent PDS was as follows: among 36 patients with PCI scores 1–10, cytoreductive surgery was associated with no residual disease in 31 (86%), residual disease measuring 0.1–1 cm in 3 (8%), and residual disease > 1 cm in 2 patients (6%). Among 20 patients with PCI scores 11–20, cytoreductive surgery was associated with no residual disease in 15 (75%), residual disease measuring 0.1–1 cm in 5 (25%), and residual disease > 1 cm in 0 patients (0%). Among 4 patients with PCI scores 21–39, cytoreductive surgery was associated with no residual disease in 1 (25%), residual disease measuring 0.1–1 cm in 3 (75%), and residual disease > 1 cm in 0 patients (0%).

Distribution of residual disease in 109 patients who underwent IDS was as follows: cytoreductive surgery was associated with no residual disease in all 13 patients (100%) with PCI scores 1–10. Among 54 patients with PCI scores 11–20, cytoreductive surgery was associated with no residual disease in 52 (96%), residual disease measuring 0.1–1 cm in 1 (2%), and residual disease > 1 cm in 1 patient (2%). Among 42 patients with PCI scores 21–39, cytoreductive surgery was associated with no residual disease in 36 (86%), residual disease measuring 0.1–1 cm in 4 (10%), and residual disease > 1 cm in 2 patients (5%).

The surgical complexity score in 60 patients who underwent PDS was as follows: among 36 patients with PCI scores 1–10, high-complexity surgery (score of 8–18) was performed in 14 (39%), moderate-complexity surgery (score of 4–7) in 15 (42%), and low-complexity surgery (score of 1–3) in 7 patients (19%). Among 20 patients with PCI scores 11–20, high-complexity surgery was performed in 17 (85%) and moderate-complexity surgery in 3 patients (15%). All 4 patients (100%) with PCI scores 21–39 underwent high-complexity surgery.

The surgical complexity score in 109 patients who underwent IDS was as follows: among 13 patients with PCI scores 1–10, high-complexity surgery was performed in 9 (69%) and moderate-complexity surgery in 4 patients (31%). Among 54 patients with PCI scores 11–20, high-complexity surgery was performed in 50 (93%), moderate-complexity surgery in 3 (6%), and low-complexity surgery in 1 patient (2%). Among 42 patients with PCI scores 21–39, high-complexity surgery was performed in 38 (90%), moderate-complexity surgery in 2 (5%), and low-complexity surgery in 2 patients (5%).

### Survival analysis

After a median follow-up duration of 40 months (range, 0.8–127 months), the median PFS for the entire cohort was 28 months, and the median overall survival (OS) was not reached.

**Figure 2:**
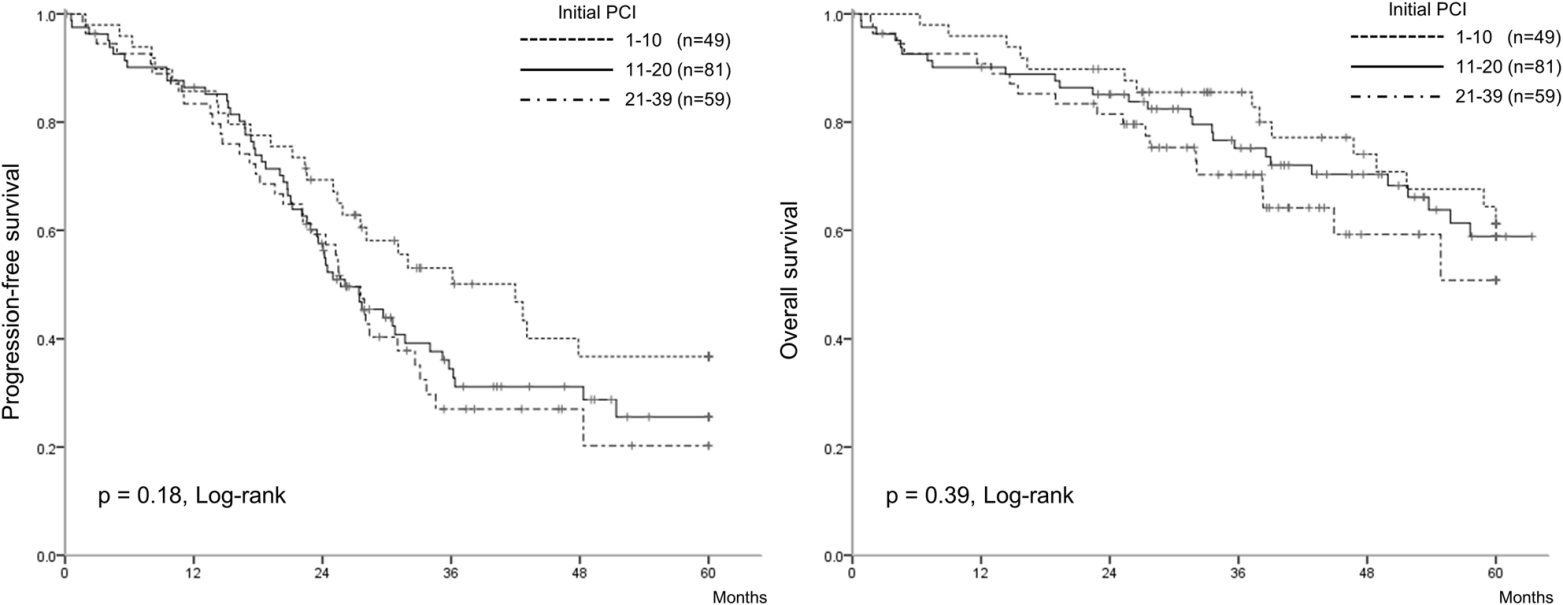
The Kaplan–Meier curves classified the patients into three groups, according to the initial PCI scores of 1–10 (n = 49), 11–20 (n = 81), 21–39 (n = 56). These PCI groups did not significantly differ in terms of progression-free survival (*p* = 0.18) and overall survival (*p* = 0.39). PCI: peritoneal cancer index.

**Figure 3:**
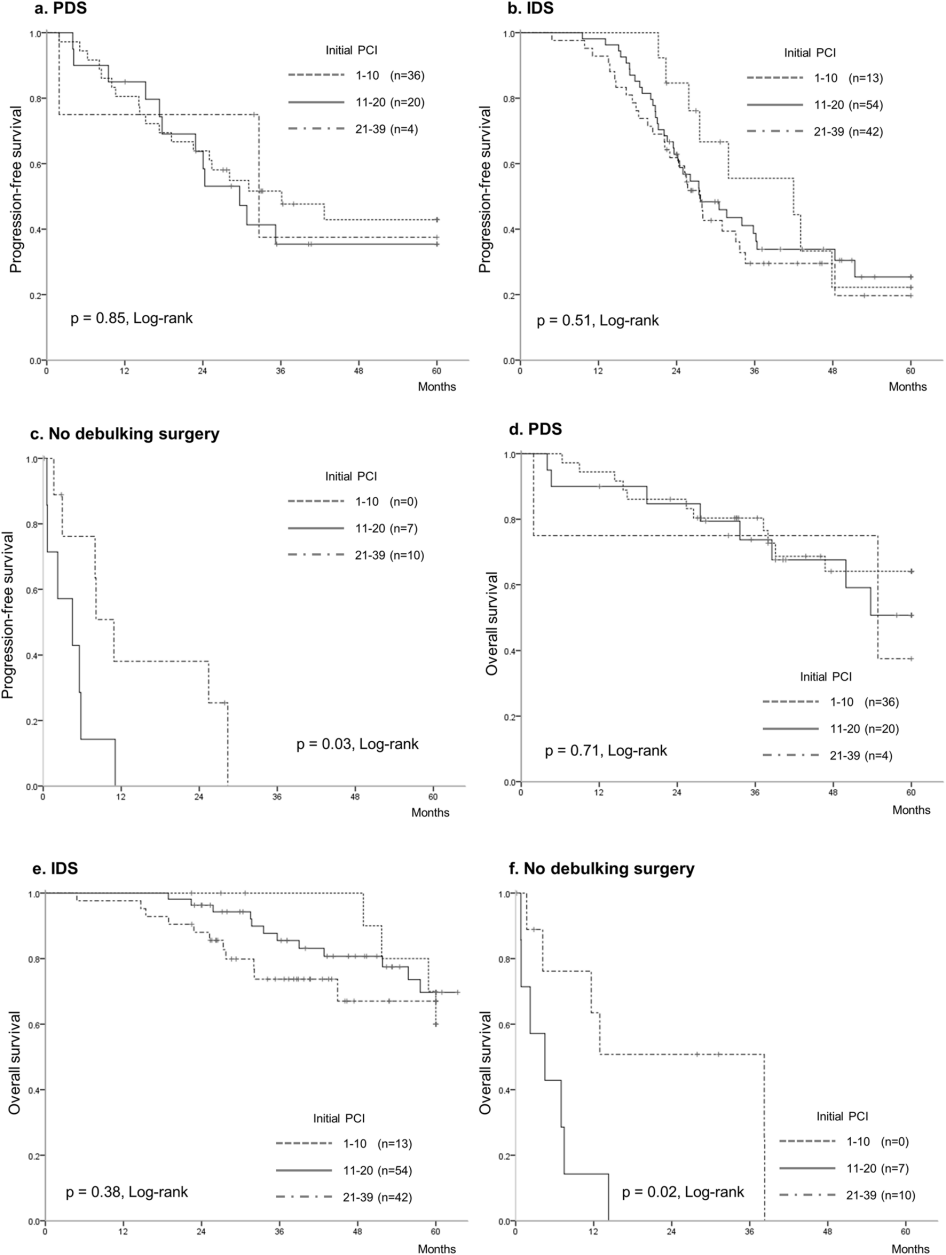
The Kaplan–Meier curves for subgroup analyses of patients who underwent PDS, for those who underwent IDS, and for those who received chemotherapy without debulking surgery. (**a**, **b**, **c**) progression-free survival. (**c**, **d**, **e**) overall survival. (**a**, **c**) patients who underwent PDS. (**b**, **d**) patients who underwent IDS. (**c**, **e**) patients who received chemotherapy without debulking surgery (no debulking surgery). Kaplan–Meier curves showed that PCI was not associated with progression-free survival and overall survival in both subgroups of patients who underwent PDS and those who underwent IDS. In contrast, PCI was associated with progression-free survival and overall survival in subgroup of patients who received chemotherapy without debulking surgery. PDS: primary debulking surgery, IDS: interval debulking surgery, PCI: peritoneal cancer index.

In the Kaplan–Meier analysis (Fig. 2), PFS and OS did not significantly differ among the initial PCI groups, which were classified into three groups based on the PCI scores: 1–10 (n = 49), 11–20 (n = 81), 21–39 (n = 56) (*p* = 0.18 and *p* = 0.39, respectively). Kaplan–Meier curves were drawn for subgroup analyses of patients who underwent PDS, for those who underwent IDS, and for those who received chemotherapy without IDS (no debulking surgery). We observed that the PCI was not associated with PFS and OS in the subgroups of patients who underwent PDS and IDS (Fig. 3a,b,d,e). In contrast, the PCI was associated with PFS and OS in the subgroup of patients who received chemotherapy without IDS (Fig. 3c,f). The PDS, IDS, and no debulking surgery groups had a median PFS of 33, 28, and 6 months, respectively, and a median OS of not reached, 72, and 8 months, respectively.

**Figure 4:**
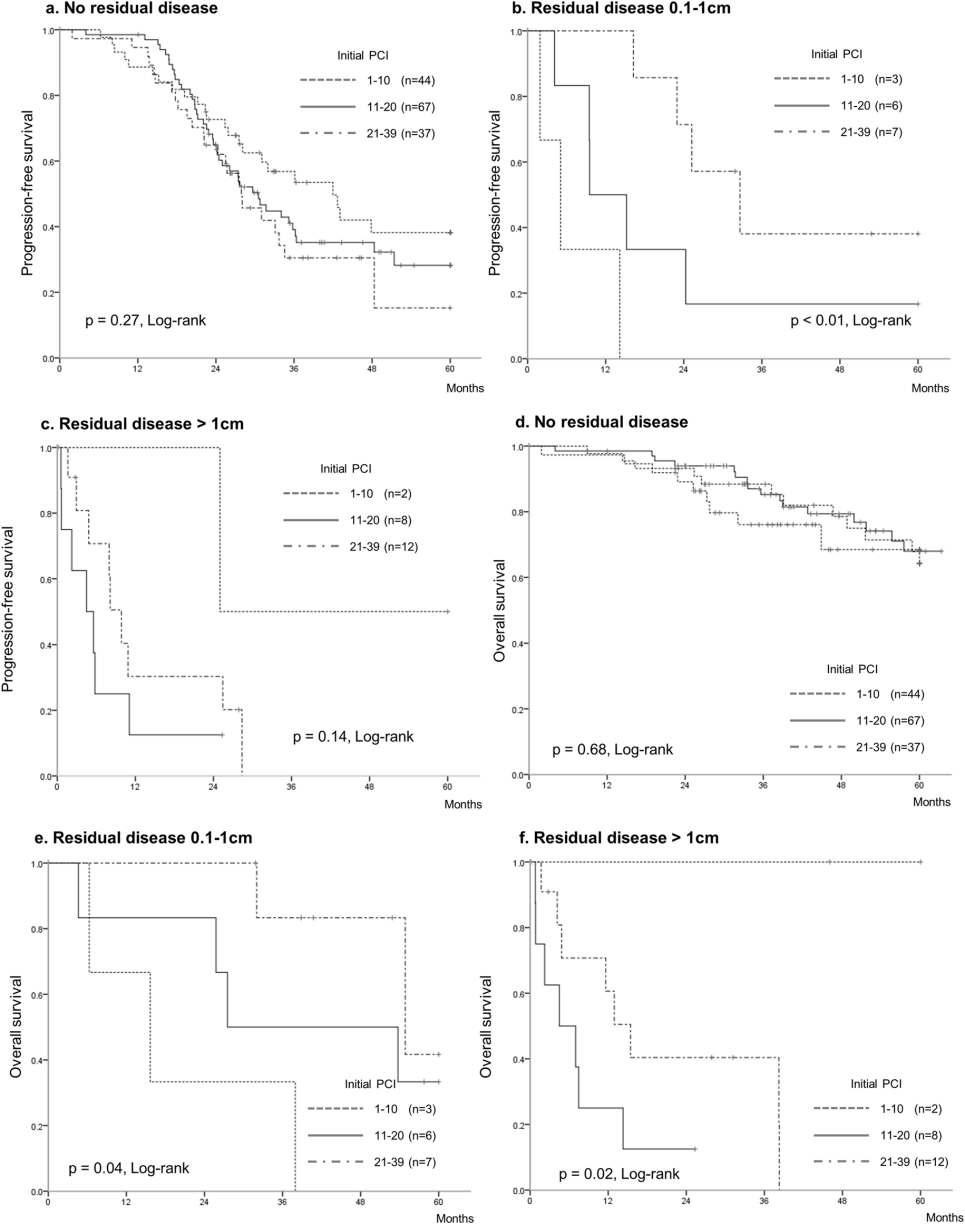
The Kaplan–Meier curves in each in each subgroup of patients with no residual disease, patients with residual disease 0.1–1 cm, and patients with residual disease > 1 cm. (**a**, **b**, **c**) progression-free survival. (**c**, **d**, **e**) overall survival. (**a**, **c**) patients with no residual disease. (**b**, **d**) patients with residual disease 0.1–1 cm. (**c**, **e**) patients with residual disease > 1 cm. Kaplan–Meier curves showed that PCI was not associated with progression-free survival and overall survival in subgroups of patients without residual disease. In contrast, PCI was associated with overall survival in patients with residual disease. PCI: peritoneal cancer index.

Kaplan–Meier curves were drawn for subgroup analyses of patients without and with residual disease (those with lesions measuring 0.1–1 cm and also those with lesions measuring > 1 cm). We observed that the PCI was not associated with PFS and OS in patients without residual disease (Fig. 4a–d). In contrast, the PCI was associated with OS in patients with residual disease (Fig. 4e,f).

Univariate Cox regression analysis showed that the initial PCI was not significantly associated with the PFS (hazard ratio [HR] 1.83, 95% confidence interval [CI] 0.83–4.09, *p* = 0.13) and with the OS (HR 2.43, 95% CI 0.87–6.86, *p* = 0.09) (Table 2). Univariate analysis showed that absence of residual disease and high surgical complexity scores significantly prolonged the PFS (HR 0.36, 95% CI 0.24–0.57, *p* < 0.01 and HR 0.62, 95% CI 0.42–0.94, *p* = 0.02, respectively) and the OS (HR 0.23, 95% CI 0.14–0.39, *p* < 0.01, and HR 0.42, 95% CI 0.26–0.70, *p* < 0.01, respectively).

**Table 2:** Univariate and multivariate analysis for progression-free survival and overall survival by the Cox regression analysis.

Variables	Univariate analysis			Multivariate analysis		
	Hazard ratio	(95%CI)	*p* value	Hazard ratio	(95%CI)	*p* value
**(a) Cox regression analysis on progression-free survival**						
PCI score^a^	1.83	(0.83–4.09)	0.13	1.83	(0.74–4.52)	0.19
Age^a^	1.01	(0.99–1.02)	0.24	1.03	(0.40–2.67)	0.95
FIGO stage						
IV versus III	1.00	(0.69–1.48)	0.96	1.07	(0.71–1.60)	0.73
Histology						
Non high-grade serous versus High-grade serous	0.91	(0.55–1.45)	0.71	0.96	(0.56–1.59)	0.89
Surgical outcome						
No residual disease versus Residual disease	0.36	(0.24–0.57)	< 0.01	0.44	(0.28–0.73)	< 0.01
Surgical complexity score						
High versus Low, Moderate	0.62	(0.42–0.94)	0.02	0.61	(0.38–1.00)	0.05
**(b) Cox regression analysis on overall survival**						
PCI score^a^	2.43	(0.87–6.86)	0.09	3.24	(0.98–10.6)	0.05
Age^a^	1.03	(1.00–1.05)	0.02	1.89	(0.53–6.90)	0.33
FIGO stage						
IV versus III	0.92	(0.56–1.50)	0.74	0.85	(0.50–1.43)	0.54
Histology						
Non high-grade serous versus High-grade serous	1.08	(0.58–1.91)	0.78	1.39	(0.69–2.62)	0.34
Surgical outcome						
No residual disease versus Residual disease	0.23	(0.14–0.39)	< 0.01	0.34	(0.19–0.63)	< 0.01
Surgical complexity score						
High versus Low, Moderate	0.42	(0.26–0.70)	0.001	0.43	(0.23–0.79)	< 0.01

PCI: Peritoneal Cancer Index, FIGO: International Federation of Gynecology and Obstetrics.

^a^Treated as a continuous variable.

Multivariate Cox regression analysis (Table 2) confirmed that the initial PCI was not significantly associated with the PFS (HR 1.83, 95% CI 0.74–4.52, *p* = 0.19) and with the OS (HR 3.24, 95% CI 0.98–10.6, *p* = 0.05). Multivariate analysis showed that absence of residual disease significantly prolonged the PFS (HR 0.44, 95% CI 0.28–0.73, *p* < 0.01), and absence of residual disease and a high surgical complexity score significantly prolonged the OS (HR 0.34, 95% CI 0.19–0.63, *p* < 0.01, and HR 0.43, 95% CI 0.23–0.79, *p* < 0.01, respectively).

## Discussion

This study showed that PCI did not affect the prognosis of patients with FIGO stage IIIC/IV ovarian, fallopian tube, and peritoneal carcinoma when initially disseminated lesions > 1 cm in diameter were resected, regardless of the timing of cytoreductive surgery. No residual disease after surgery and high-surgical complexity score affected the prognosis. In other words, aggressive surgery could overcome the extent of peritoneal dissemination when patients with a high PCI score undergo high-complexity surgery by selecting PDS or IDS, depending on whether cytoreduction to no residual disease is achievable at initial laparotomy before starting treatment.

In this study, the timing of cytoreductive surgery was determined at initial laparotomy on the based on the feasibility of achieving no residual disease. As a result, the number of the patients who did not underwent PDS was more than twice as large as the number of the patients underwent PDS. Contrary to our study, the timing of cytoreductive surgery was usually primary at many institutions where aggressive cytoreductive surgery was performed^16–19^. However, studies have shown that the PCI score was negatively associated with the completeness of cytoreduction and residual disease after PDS^10^. Moreover, the rate of complications with PDS was higher than that with IDS^18,24^. Conversely, the use of NACT/IDS enabled the surgeons to accomplish a safe and high-complexity surgery in patients with high extent of peritoneal dissemination because NACT reduces the massive ascites and the tumor volume, improves the general condition, and decreases the difficulty of complex surgery. In this study, high-complexity surgery was performed on 71% of the entire cohort; this led to high rate (80%) of complete cytoreduction rate. Our results showed that not only PDS but also selective use of NACT/IDS allowed many patients to receive complete cytoreduction safely, regardless of the initial PCI.

In this study, the surgical complexity score of the patients who underwent IDS was higher than those of the patients who underwent PDS because initial PCI of the patients who underwent IDS was higher than those of the patients who underwent PDS. On the contrary, several studies showed that the surgical complexity and the rate of extra-gynecologic surgery were lower with IDS than with PDS^18,25,26^. The difference between this study and others lies in the surgical policy for IDS. In the present study, all disseminated tumors > 1 cm in diameter identified at initial laparotomy was removed even if the initially disseminated tumor was no longer visible at the time of IDS after NACT. In other words, at IDS, we performed the surgical procedures that would be performed to achieve residual tumor < 1 cm if PDS were to be performed. A drawback of the conventional IDS is that the tumors > 1 cm diameter identified at initial laparotomy would not be resected during IDS if they become invisible due to a good response to NACT. This may lead to a risk of early recurrence or relapse, similar to residual disease > 1 cm diameter after PDS. Our previous study showed that microscopic disease remains present especially in the rectosigmoid colon, transverse mesentery, greater omentum, right diaphragm, paracolic gutters, and vesicouterine pouch even if the tumors become invisible during IDS^20^. Similar to ours, Lim et al. reported that detached scars had residual cancer cells that assumedly included cancer stem cells^27,28^. The surgical removal of all the tumors > 1 cm identified at initial laparotomy could overcome tumor biology and tumor burden.

Unlike gastrointestinal carcinoma, another reason that PCI did not affect prognosis in this study is the high response rate to chemotherapy for ovarian cancer. In this study, 87% patients did not have disease progression during NACT and could undergo IDS. In other reports, the response rates to chemotherapy in FIGO stage III/IV ovarian carcinoma is higher in colorectal carcinoma (73–90 vs 15–62%)^18,29–32^. For colorectal cancer, patients with high score of PCI were reported to have poor prognosis^29,33–36^ because PCI directly affect the rate of no residual disease after surgery, frequency of postoperative complications, and prognosis^29,33–36^. Similar results have been reported for gastric and appendix cancers.

This study had some limitations such as the single-institution analysis, the small number of patients, and a higher percentage of IDS cases compared with that of PDS cases. The selection criteria for performing PDS or IDS varies among institutions. Notably, 42 of 56 patients with a high PCI score (21–39) were included in the NACT/IDS group; therefore, it is reasonable to conclude that in addition to aggressive surgery, chemotherapy may have affected the prognosis. In this study, 73% of patients with a high PCI score who could not undergo PDS underwent high-complexity surgery following the administration of NACT. Therefore, optimal patient selection for PDS or IDS can enable high-complexity surgery with favorable prognosis even in patients with a high PCI score.

In conclusion, the extent of initial peritoneal dissemination did not affect the prognosis for patients with FIGO stage IIIC/IV ovarian, fallopian, and peritoneal carcinoma when initially disseminated lesions > 1 cm was resected, regardless of the timing of cytoreductive surgery. No residual disease after surgery and a high-surgical complexity led to favorable prognosis. Aggressive cytoreductive surgery with selective use of IDS could overcome the extent of peritoneal dissemination.

## Methods

### Patient selection

This study was approved by the Institutional Review Board of Chiba University Graduate School of Medicine. All methods were carried out in accordance with relevant guidelines and regulations. Patients with FIGO 2014 stage IIIC/IV ovarian, fallopian tube, and peritoneal carcinoma who were consecutively treated at Chiba University Hospital from January 2008 to December 2017 were included. Written informed consent was obtained from all patients before surgery. Of the 208 patients during the study period, 22 patients who did not undergo exploratory laparotomy before NACT because of poor general condition (performance status ≥ 3 or ileus) and/or those aged ≥ 80 years, or who did not be evaluated in whole abdominal cavity due to adhesion were excluded. Therefore, this study included 186 patients in whom the extent of peritoneal dissemination upon initial laparotomy was assessed.

### Selection of Primary or Interval debulking surgery

The treatment protocol used in this study is described in our previous reports^20,21^. In brief, the selection of primary or interval debulking surgery was decided during the initial laparotomy which was performed as early as possible after a patient's first visit. The patients was selected for IDS when disseminated tumor burden explored at initial laparotomy was too high to achieve no residual disease; gastrectomy, resection of the hepatic hilum or head of the pancreas, massive intestinal resection, or total colectomy was required; and/or massive ascites caused coagulopathy. At that time, patients underwent ovarian, fallopian tube, or omental biopsies, followed by NACT. Patients other than those mentioned above were selected for primary debulking surgery including upper abdominal surgery and bowel resection, to achieve no residual disease after surgery.

### The exploration of initial peritoneal dissemination

The extent of peritoneal dissemination was assessed during initial laparotomy according to the peritoneal cancer index (PCI)^1,2^. The initial PCI groups were classified into three groups based on the PCI scores: 1–10, 11–20, 21–39. When the patients were selected for IDS, the margins of the disseminated tumor (> 1 cm) were marked with a non-absorbable, 3–0 black silk suture after diagnostic biopsy of ovary, fallopian tube or omentum was performed^20,21^.

### Interval debulking surgery

The timing of IDS was described at our previous reports^20,21^. An implantable port system placed in the abdominal cavity at initial laparotomy was used for collection of peritoneal washing cytology, which was performed every 3 to 4 weeks during NACT. IDS was performed when the peritoneal washing cytology was negative and/or the serum CA-125 had decreased to 15 IU/mL or the serum CA-125 level stopped decreasing when the peritoneal washing cytology remained positive. If the disease progressed during NACT, we did not perform IDS. During IDS, the regions in which the disseminated tumors > 1 cm at the initial laparotomy was removed even when initial tumors were invisible, using the non-absorbable suture marked at initial laparotomy as landmarks.

### The complexity of surgery performed

The complexity of surgery performed were scored according to the surgical complexity score^22,23^. The surgical complexity score was classified into three groups: the score of 1–3 was low, 4–7 was moderate, and 8–18 was high^22,23^.

### Chemotherapy

For the adjuvant chemotherapy after PDS, six cycles of weekly paclitaxel (60–80 mg/m^2^) and carboplatin (AUC 2–3) for 3 weeks were administered. For the NACT, weekly paclitaxel and carboplatin were administered until the conditions mentioned above^20^. The median number of NACT cycles was 5. For adjuvant chemotherapy after IDS, six cycles of gemcitabine (500 mg/m^2^) and irinotecan (50 mg/m^2^) on days 1 and 8 every 3 weeks were administered. After December 2013, upon its approval for use by the Japanese public insurance, bevacizumab (15 mg/kg) every 21 days for 21 cycles was administered as the first-line therapy.

### Statistical analysis

The association between the initial PCI and the clinical factors were analyzed using the Mann–Whitney *U*-test and Kruskal–Wallis *H*-test. Age and initial PCI score were entered as continuous variables. The association between the initial PCI and survival was analyzed by the Kaplan–Meier. Kaplan–Meier curves were drawn for the entire cohort and for subgroups of patients who underwent PDS, for those who underwent IDS, and for those who received chemotherapy without IDS. Kaplan–Meier curves were also drawn for subgroups of patients without and with residual disease (those with lesions measuring 0.1–1 cm and also those with lesions measuring > 1 cm). Univariate and multivariate Cox proportional hazards regression analyses were performed to analyze the prognostic factors. All statistical analyses were performed using JMP ver. 11(SAS, Cary, NC, USA). The Kaplan–Meier survival curves were drawn using IBM SPSS Statistical ver. 22 (IBM Japan Services Co., Ltd., Tokyo, Japan). For all statistical tests, the differences were considered significant at *p* < 0.05.
